# Creating a Real-World Linked Research Platform for Analyzing the Urgent and Emergency Care System

**DOI:** 10.1177/0272989X221098699

**Published:** 2022-05-14

**Authors:** Suzanne Mason, Tony Stone, Richard Jacques, Jennifer Lewis, Rebecca Simpson, Maxine Kuczawski, Matthew Franklin

**Affiliations:** School of Health Related Research, University of Sheffield, Sheffield, England, UK; School of Health Related Research, University of Sheffield, Sheffield, England, UK; School of Health Related Research, University of Sheffield, Sheffield, England, UK; School of Health Related Research, University of Sheffield, Sheffield, England, UK; School of Health Related Research, University of Sheffield, Sheffield, England, UK; School of Health Related Research, University of Sheffield, Sheffield, England, UK; School of Health Related Research, University of Sheffield, Sheffield, England, UK

**Keywords:** data linkage, emergency care, health data, research-ready data, routine data, routine data analysis, urgent care

## Abstract

**Background:**

This article describes the development of a system-based data platform for research developed to provide a detailed picture of the characteristics of the Urgent and Emergency Care system in 1 region of the United Kingdom.

**Data Set Development:**

CUREd is an integrated research data platform that describes the urgent and emergency care system in 1 region of the United Kingdom on almost 30 million patient contacts within the system. We describe regulatory approvals required, data acquisition, cleaning, and linkage.

**Data Set Analyses:**

The data platform covers 2011 to 2017 for 14 acute National Health Service (NHS) Hospital Trusts, 1 ambulance service, the national telephone advice service (NHS 111), and 19 emergency departments. We describe 3 analyses undertaken: 1) Analyzing triage patterns from the NHS 111 telephone helpline using routine data linked to other urgent care services, we found that the current triage algorithms have high rates of misclassifying calls. 2) Applying an algorithm to consistently identify avoidable attendances for pediatric patients, we identified 21% of pediatric attendances to the emergency department as avoidable. 3) Using complex systems analysis to examine patterns of frequent attendance in urgent care, we found that frequent attendance is stable over time but varies by individual patient. This implies that frequent attendance is more likely to be a function of the system overall.

**Discussion:**

We describe the processes necessary to produce research-ready data that link care across the components of the urgent and emergency care system. Making the use of routine data commonplace will require partnership between the collectors, owners, and guardians of the data and researchers and technical teams.

**Highlights:**

- This article describes the development of a system-level data platform for research using routine patient-level data from the urgent and emergency care system in 1 region of the United Kingdom.
- The article describes how the data were acquired, cleaned, and linked and the challenges faced when undertaking analysis with the data.
- The data set has been used to understand patient use of the system, journeys once in the system, and outcomes following its use, for example, patterns of frequent use within urgent care and accuracy of referral decisions within the system.

## Background

Research using depersonalized routine health and social care data can provide unique insights to improve population health and well-being. There is a wealth of routine health and social care data from real-world settings such as hospitals, primary care, and local authorities that have important secondary uses such as research. However, because of challenges in linking and sharing these data sets, their potential to enable powerful, efficient research that informs health policy and services is not being realized.

Advantages to using depersonalized routine data sets for research include their large size and real-world nature, which is more representative of populations and service delivery. Information is captured about groups who are typically underrepresented in research, such as older people with multiple medical problems and vulnerable groups such as migrants or homeless people. The ability to link between data sets further improves the accuracy and completeness of the data available for research. In addition, using existing data minimizes the cost and logistical challenges of data collection in research. As stated in the UK Life Sciences Vision,^1^ Life Sciences Industrial Strategy,^2^ and National Institute for Health and Care Research Best Research for Best Health,^3^ unlocking the potential of real-world data presents huge opportunities for research to develop solutions addressing inequalities within populations where risk is high and access to care poorer.

Urgent and emergency care (UEC) services provide substantial health benefits across the world, but increasing demand is leading to unsustainable pressure on services and the need for health care funding. In the English National Health Service (NHS) in 2018–2019, there were 24.8 million attendances at major emergency departments (EDs), single specialty EDs, walk-in centers, and minor injury units, at a cost of £2.1 billion; 5.3 million emergency hospital admissions, 7 million ambulance service journeys; and approximately 24 million calls to NHS UEC telephone services (NHS 111).^4^ Failure of the UEC system to manage increasing demand causes substantial public concern and political impact.

There is a lack of data and analytical capabilities to provide a detailed picture of the characteristics of the whole UEC system, including describing and understanding demand, variation in pathways of care and patient outcomes across telephone helplines, ambulances, Eds, and acute hospital admissions. Individual provider data exist, such as ambulance and ED, but there have been limited attempts to link data across different providers to show patient flow through the whole system across large populations, to understand how the system is used from the point of contact (such as a call to the emergency ambulance service [999] or the national telephone helpline [NHS 111]) through different parts of the system (into ED and into hospital). The ability for researchers to harness and link together these data is key to understanding how the system is functioning and therefore how and where to develop appropriate patient-focused interventions that can lead to a sustainable, safe, and cost-effective system of care.

This article describes how we have developed an integrated research data platform for the UEC system in a large region of the United Kingdom. We have called this research platform CUREd (www.sheffield.ac.uk/scharr/research/centres/cure/projects/cured-how-access-data). The platform contains several data sets, all of which cover the geographical area of Yorkshire and the Humber (YH), United Kingdom. YH has a population of 5.6 million and a mixed urban, suburban, and rural geography. The population is ethnically diverse and contains areas of severe deprivation and multimorbidity as well as affluent areas and with fewer health challenges.^5^ Data are linked to trace the UEC system from the patient call through to discharge from the system or death and covers a period from 2011–2017 for 14 acute NHS Trusts, 1 ambulance service, the NHS 111 national telephone helpline, and 19 EDs. Data are analyzed in order to track and describe patient journeys, interactions, and outcomes within this system. It can identify variation in demand, access, and outcomes and also where outcomes and provision need to be improved for certain patient groups, localities, and services.

This article outlines the development of the CUREd data research platform and how it is currently being used for the delivery of applied health research and knowledge translation work in the field of UEC.

## Data Set Development

The rationale for developing this data set came from the need to have access to research-ready real-world data for multiple research purposes. Data exist in the United Kingdom from NHS sources such as NHS Digital; however, our experience was that such data were time-consuming to obtain with many obstacles for research teams to navigate. We set objectives to develop our data set for the purposes of researching the UEC system in 1 region as part of a funded project, Connected Health Cities (https://www.connectedhealthcities.org/), aiming to connect health care systems and data across areas of the North for research leading to improving health and well-being in this region of the United Kingdom.

We have been unable to identify similar research databases in the United Kingdom that have approvals in place through the NHS National Health Research Authority.^6^ The 2 databases we identified were our own iterations of the data platform described in this article.

### Regulatory Approvals and Governance Arrangements

Prior to accessing potentially identifiable health data in the United Kingdom, researchers are required to evaluate and justify their use from both ethical and regulatory perspectives. Obtaining the data necessary to build the CUREd platform required a number of challenges to be overcome.

The CUREd platform would contain millions of patients who had used 1 or more UEC service over a 5-y period in the YH region; thus, it was recognized that obtaining direct patient consent would be impractical because of the limited resources available for developing the data set. In addition, it would be expected that many patients would have died or otherwise lack the capacity to consent due to the severity of their illness or injury, potentially leading to an underrepresentation of these groups of patients within the data and thereby limiting its value.

In making our application for data access, we took an “opt-out” approach, whereby identifiable patient data were included but patients had the option to have their details removed if they notified us. Support to use this consent model was sought from the NHS Confidentiality Advisory Group (CAG), an independent body within the NHS Health Research Authority, which provides expert advice on the use of confidential patient information within England and Wales. Following review, a favorable opinion was provided by the National Research Ethics Service (18/YH/0234) and CAG, the NHS Health Research Authority granted an exemption to the common law duty of confidentiality under section 251 of the NHS Act 2006 (18/CAG/0126), providing a legal basis for data sharing for essential medical research. CAG granted conditional approval on the basis that a poster was placed in all UEC services contributing to the CUREd platform in patient-facing areas detailing the opt-out process they could follow. The study website contains information for participants, how they can have their details removed from the database, and information on data security. To date, no patients have made a request to withdraw from the study.

A Data Release Committee (DRC) was formed as an oversight panel for the CUREd platform. The panel includes patient and public representation, health care stakeholders, and information governance specialists. The DRC reviews all applications to access the data by researchers and ensures all data releases are appropriate in terms of the study, variables requested, risk (such as potential for reidentification), and information governance in place. A public register of approved CUREd platform data releases is available on the study website.

### Acquisition, Preparation, and Linkage of the Data

#### Data acquisition

Data were requested from 14 NHS Acute Trusts (responsible for 19 EDs) and 1 ambulance service that provide both ambulance response (999) and telephone helpline (NHS 111) services to the geographical area of Yorkshire and Humber for the period April 1, 2011, to March 31, 2017 (inclusive). The start date was based on the number of years of consistent data that Trusts could extract from their information systems without excessive effort. The end date was chosen to align to the end of the NHS reporting year for the most recent complete year at the time the data request was made. The data specification sent to NHS Acute Trusts specified data coded using nationally defined coding standards. The ED and inpatient data sets specified consisted of 69 and 115 fields, respectively. Each field required validation; this was accomplished through pattern-matching rules or validation against code sets. Data quality varied (e.g., the omission of specified variables, the use of local code values rather than specified national codes, corruption of values, for example, by the omission of meaningful leading zeros) along with the time taken to supply data. Further attempts were made to obtain missing or corrected items from organizations. A data dictionary detailing all variables available from the CUREd database is published on the project website.

#### Data linkage

Many examples in the health data linkage literature seek to identify only a single cohort from patient records appearing in 1 source (“master”) or all sources (“nested” or “intersectional”).^4,7^ This project aimed to identify individuals (entities) across all data sources (“union”) in the absence of any “master” source. We used a combination of deterministic and probabilistic record linkage techniques.

##### Deterministic entity resolution

The presence of common, high-quality, highly discriminatory identifiers (e.g., NHS number) makes deterministic linkage as valid as probabilistic linkage.^8^ A valid NHS number was recorded for 99.7% of admitted patient care (inpatient) episodes, 98.0% of ED attendances; 96.3% of NHS 111 calls, and 14.4% of ambulance incidents. As deterministic methods are considerably less computationally expensive than probabilistic methods, a first deterministic entity resolution step was employed among inpatient, ED, NHS 111, and ambulance records. The term *entity resolution* is used here because the aim was not to simply link one set of data to another set of data but rather to identify all records amongst the data sets that correspond to the same individual (see Box 1). Table 1 demonstrates the success of data linkage using deterministic methods.

**Box 1:** Process of Deterministic Entity Resolution

Step 1: Assign each distinct pair (valid NHS number, valid date of birth, a distinct CUREd identifier (CUREd ID) Step 2: Attempt to link records with valid NHS numbers but no valid date of birth to a CUREd ID based on approximate birth year (calculated from activity date and age at activity) Step 3: Attempt to link remaining records to an assigned CUREd ID by provider code, provider patient ID, and date of birth matches (provided this matches only 1 CUREd ID)^1^ Step 4: Attempt to link remaining records to an assigned CUREd ID by first name, last name, sex, date of birth, and postcode matches (provided this matches only 1 CUREd ID)^2^ Step 5: Attempt to link remaining records to an assigned CUREd ID by sex, date of birth, and postcode matches (provided this matches only 1 CUREd ID)^3^ Step 6: Cluster remaining records by agreement on any of the following patterns: 1. Provider code, provider patient ID, and date of birth matches^1^ 2. First name, last name, sex, date of birth, and postcode^2^ 3. Sex, date of birth, and postcode matches^3,4^ and assign each distinct cluster to a new CUREd ID Step 7: Assign each remaining record to its own CUREd ID 1. Ambulance records excluded as no provider patient ID was available 2. NHS 111 helpline records were excluded as names were not available 3. We excluded 1% of postcodes with greatest number of distinct patients registered at such postcodes. These likely represent communal establishments, such as prisons. 4. Ambulance records excluded as recorded postcodes related to incident locations rather than place of residence

^1^Ambulance records excluded as no provider patient ID was available^2^NHS 111 helpline records were excluded as names were not available^3^We excluded 1% of postcodes with greatest number of distinct patients registered at such postcodes. These likely represent communal establishments, such as prisons.^4^Ambulance records excluded as recorded postcodes related to incident locations rather than place of residence

**Table 1:** Number and Percentage of Records Assigned to a CUREd ID at Each Deterministic Entity Resolution Step for Each Source Data Set^a^

Number (*N*) (Column, Percentage)	Source Data Set				All Data Sets
	NHS 111 Telephone Helpline	Ambulance Service	Emergency Department	In-Patient Admissions	
Total records	4,789,273	4,382,835	9,822,644	10,308,510	29,303,262
NHS number, date of birth	4,610,299 (96.3%)	629,315 (14.4%)	9,628,629 (98.0%)	10,274,792 (99.7%)	25,143,035 (85.8%)
Local patient ID and date of birth link to NHS number	18,153 (0.4%)	N/A (0.0%)	21,659 (0.2%)	1079 (<0.1%)	40,891 (0.1%)
Name, sex, date of birth, and postcode link to NHS number	N/A (0.0%)	688,520 (15.7%)	3411 (<0.1%)	785 (<0.1%)	692,716 (2.4%)
Sex, date of birth, and postcode link to NHS number	22,291 (0.5%)	N/A (0.0%)	31,756 (0.3%)	1177 (<0.1%)	55,224 (0.2%)
Clustered	138,282 (2.9%)	40,634 (0.9%)	133,572 (1.4%)	17,776 (0.2%)	330,264 (1.1%)
No deterministic link/cluster possible	248 (<0.1%)	3,024,366 (69.0%)	3617 (<0.1%)	12,901 (0.1%)	3,041,132 (10.4%)

a Probabilistic record linkage: ambulance and emergency department/inpatient record. NHS, National Health Service.

##### Probabilistic record linkage: ambulance and emergency department/inpatient record. NHS, National Health Service

A valid NHS number was recorded for only 14.4% of ambulance incidents; thus, for the ambulance records, a further linkage step was required. A total of 60.1% of ambulance incidents resulted in conveyance to a hospital; thus, a matching contemporary ED attendance or inpatient admission record could be expected. This provided a means to link ambulance records to ED and inpatient records. We used probabilistic record linkage techniques^9–11^ based on work originally proposed by Fellegi and Sunter.^12^ To reduce the search space from almost one hundred thousand billion comparisons, we used (deterministic)

1. “blocking” using the (hospital) site to which the patient was conveyed
2. “windowing” using the date and time to create ranges of interest

We used additional blocking strategies to reduce the still considerable search space further by requiring at least 1 of the following patterns:

1. postcode match
2. first letter of first name match and first letter of last name match
3. first letter of first name match and age differs by ≤10 y
D. first letter of last name match and age differs by ≤10 y
E. Date of birth differs by ≤31 d
F. Date of birth year match and date of birth month and day transposed
G. Date of birth month and date of birth day match

The windowing was specified such that ambulance records were compared only with the following:

- ED records that had an ED arrival date time up to 1 h before the ambulance arrival at conveyance destination date time and up to 3 h after that time AND
- inpatient records (for which only dates, not times, are reliably recorded) recorded as admission episodes that occurred on the same day AND
  - if the ambulance arrival at the conveyance destination date time was before 1 a.m.: inpatient admission episode records on the previous day or
  - if the ambulance arrival at the conveyance destination date time was after 9 p.m.: inpatient admission episode records on the following day

The following 8 fields were compared:

- year of birth
- month of birth
- day of birth
- age (at time of activity)
- first name (string difference using Jaro-Winkler algorithm)
- last name (string difference using Jaro-Winkler algorithm)
- postcode
- sex

Each comparison of record pairs resulted in 1 of 28 possible binary agreement patterns (e.g., agreement or disagreement on each of the compared fields). Agreement and disagreement weights for each compared field were calculated from the parameter estimates from the expectation-maximization algorithm as implemented in the RecordLinkage package for R.^13^ This algorithm is based on a latent class model for the compared record pairs in which one class is the set of true matches and the other the set of true nonmatches. The algorithm iteratively estimates, for each compared field, 1) the probability of agreement on that field when the comparison pair is a match and 2) the probability of agreement when the comparison pair is a nonmatch, such that these parameters maximize the likelihood of observing the (observed) agreement pattern frequencies. The weights give the relative distinguishing power of the compared fields with respect to each other. Agreement weights for first name and last name fields, where compared values were similar but did not agree perfectly, were adjusted downward using the Jaro-Winkler string comparator algorithm.^14^ This algorithm provides a measure of similarity between 2 pieces of text, based on the proportion of matching characters and number of transpositions of characters, with greater weight given to the early part of the texts. For each pair of records, a total match weight was calculated by summing the agreement weights for each field on which there was agreement (or, in the case of first and last name, sufficient similarity) and (negative) disagreement weights for each field on which there was disagreement.

Pairs of records with a match weight greater than or equal to a match weight threshold were considered to belong to the same patient. The match weight threshold was selected using the subset of compared records for which NHS numbers for both records in the pair were present (18.7% of pairs). Agreement on NHS number was considered to indicate a “real” match and disagreement a “real” nonmatch.

**Figure 1:**
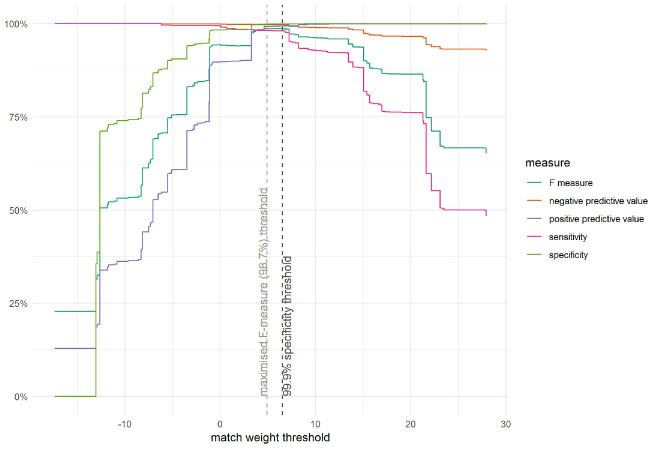
Measures of linkage performance displayed for different match weight thresholds for a subset of compared records with valid National Health Service (NHS) numbers. All links with a match weight above the threshold were considered valid; however, some ambulance records matched to more than 1 hospital record (or vice versa). In this case, only the greatest weighted link was retained and only if its weight was twice (or more) of the weight of the next greatest weighted link. For each probabilistically linked pair, both the ambulance record and the hospital record may have themselves been deterministically linked to other records. Before proceeding, it was necessary to ensure that these wider pools of linked records did not conflict with one another; that is, we needed to ensure there was no conflict in the transitivity due to the probabilistic linkage. If a conflict was detected (e.g., conflicting NHS numbers or date of births), the probabilistic link was disregarded.

Figure 1 shows a variety of binary classification performance measures across the possible match weight thresholds. A threshold could be chosen on the basis of any performance measure and for any particular value for that performance measure. The choice depends on the use case. In our study, we wished to balance sensitivity and specificity so chose a match weight threshold corresponding to the 99.9% specificity limit (i.e., a 1 in 1000 probability of a false match); this threshold corresponded to a sensitivity of 98.1%. This threshold was also close to the threshold corresponding to the maximum F-measure value (98.7%), a measure of classification performance balancing positive predictive value and sensitivity.

Table 2 demonstrates the results from the probabilistic linkage of records. Of all ambulance records that involved conveyance to the hospital, 69.1% were linked. However, for ambulance records not involving conveyance to the hospital, only 23.9% were linked. Of all ambulance records linked, 39.9% were linked using probabilistic methods.

**Table 2:** Number and Percentage of Records Assigned to a CUREd ID Using Probabilistic Linkage for Each Source Data Set

Number (Column, Percentage)	Source Data Set				All Data Sets
	NHS 111 Telephone Helpline	Ambulance Service	Emergency Department	Inpatient Admissions	
Records not deterministically linked/clustered	248	3,024,366	3617	12,901	3,041,132
Probabilistic link to record(s) with (or deterministically linked to) NHS number	4 (1.6%)	886,089 (29.3%)	851 (23.5%)	36 (0.3%)	886,980 (29.2%)
Probabilistic link deterministically clustered records	0 (0.0%)	6289 (0.2%)	0 (0.0%)	0 (0.0%)	6289 (0.2%)
No linkage	244 (98.4%)	2,131,988 (70.5%)	2766 (76.5%)	12,865 (99.7%)	2,147,863 (70.6%)

##### Duplicate detection and removal

Calls, incidents, attendances, and episodes were plotted for each data provider by month of start of call, month of attendance, and month of episode start date, respectively. These plots displayed incongruous data points, suggesting some providers had supplied duplicate records around the start of each NHS reporting year (April). We considered records duplicate if they belonged to the same patient, had the same start and finish dates and times (where applicable), and other key clinical information (such as treating specialty, primary diagnosis) was identical; in these cases, a single record from the set of duplicates was retained.

##### Final platform

Some records were excluded from the final platform based on activity date lying outside of the period or no date being present. The dates considered were: date of start of call for NHS 111 and ambulance records; arrival date for ED records; and episode start date for inpatient records. In addition, inpatient records that were recorded with an admission method other than an emergency admission were discarded. Table 3 demonstrates the final results of linkage. Records that were not “linked” were retained and each given a distinct CUREd ID. These records represent genuine care activity possibly belonging to a patient with no NHS number and not elsewhere present in the data within the period (e.g., a foreign tourist) or the result of the identifying data recorded being insufficient or too inaccurate to afford a link.

**Table 3:** Number of Records in—and Exclusions from—the Final Database for Each Source Data Set

Number	Source Data Set				All Data Sets
	NHS 111 Telephone Helpline	Ambulance Service	Emergency Department	In-Patient Admissions	
Total records	4,789,273	4,382,835	9,822,644	10,308,510	29,303,262
Duplicate records	0	0	1918	188,274	190,192
Other exclusions	0	0	33,456 (outside date range or date missing)	5,533,347 (5,527,416 nonemergency admissions; 5931 out outside of date range or date missing)	5,566,803
Final records	4,789,273	4,382,835	9,787,270	4,586,889	23,546,267

## Analyses Using the CUREd Data Set

The CUREd data have been linked in various ways to address different questions. It has also been linked with other external data sources. Examples include exploring telephone advice service (NHS 111) pathways, the characteristics of nonurgent attendances in children, and a complex systems analysis showing that frequent attendance at ED can be described using power laws.

### Telephone Advice Services (NHS 111)

The NHS 111 pathways project used the linked data of all adult NHS 111 calls, subsequent ED attendances within 48 h of the 111 call, and acute hospital admissions up to 1 night after that ED attendance. The project aimed to explore to what extent patients complied with NHS 111 recommendations and to what extent NHS 111 recommendations were triaged appropriately according to later classifications at the ED. In particular, we wished to investigate what proportion of NHS 111 calls resulting in a recommendation to the patient to self-care or consult primary care were later followed by an ED attendance, indicating noncompliance. We were further interested in how often these ED attendances were classified as “urgent,” which would imply that the original NHS 111 recommendation was insufficient. Finally, we wished to investigate what proportion of NHS 111 calls resulting in a high-acuity recommendation (whether an ambulance was sent or the patient was advised to attend the ED) further resulted in an “urgent” classification at ED^15^ and/or a subsequent hospital admission.

We found that about 10% of patients who were advised to self-care or to consult primary care actually attended the ED.^16^ This represents a considerable proportion of low-acuity NHS 111 recommendations that are not complied with and consequently a significant amount of UEC resources consumed by unadvised ED attendances. However, about 88% of these noncompliant visits were subsequently classified as urgent, suggesting that many of these patients should have received a higher-acuity recommendation from NHS 111. In addition, of high-acuity NHS 111 recommendations that were followed by patients, we found about 10% to be nonurgent and can be considered “mis-triages.”

Taken together, these results suggest that, in many cases, NHS 111 algorithms being used may be systematically misclassifying the urgency of patient concerns in both high- and low-acuity cases. These findings were discussed in the context of available health care services, highlighting that both high-accuracy algorithms and requisite health care provisions such as out-of-hours GP services must be in place for NHS 111 to be an effective triaging system.

### Avoidable Attendance at the ED

This project explored the characteristics of nonurgent attendances in children.^17^ Using a predefined definition of a nonurgent attendance,^15^ the project aimed to understand the size of the problem, which children present in this way, and when. Data were composed of deidentified ED attendances for all children (aged from 0 to 15 y) who attended a type 1 ED (consultant-led, multispecialty 24-h services with full resuscitation facilities and designated accommodation for the reception of ED patients) in Yorkshire and Humber.

For this analysis, we extracted the following variables from the data set: age, sex, date of attendance, attendance category (first or follow-up attendance), trust, arrival mode (ambulance or other), disposal (including whether discharged, admitted, or referred for follow-up), time of arrival, time to assessment, time to treatment and time to departure, department type (type 1, 2, or 3 ED), location of incident, clinical investigations, clinical treatments, and diagnosis.

ED data for children in the CUREd research database were incomplete in the period from 2011 to 2013, with missing data preventing the calculation of nonurgent attendances using our definition for a number of trusts. This meant the analysis was focused on children’s ED attendances between April 2014 and March 2017, when data were complete. Because of the data quality on presentation and diagnosis, analyses were limited, as it was not possible to case-mix adjust and explore certain subgroups of children further.

We found that the overall rate of nonurgent attendances in children was 21%. Nonurgent attendances were more likely to present in the youngest age categories, with more than half in those younger than 5 y. We also found that a high number of nonurgent attendances were during out-of-hours periods (in hours defined as 0800–1800 h Monday–Friday). Finally, the nonurgent attendances in the youngest age group were more likely to arrive by ambulance compared with those older than 5 y. The results from this project suggest that children younger than 5 y would be a group who would potentially benefit from targeted interventions, such as providing accessible care out of hours.

### ED Attendance Patterns

Attendance patterns at EDs among frequent attenders were examined using a complex systems analysis approach.^18^ This demonstrated that ED attendance patterns can be consistently described using power laws, which identify that there are, at any given time, a relatively small number of very-high ED users. These patterns remained stable over time, despite the fact that individual frequent attenders in a given year were often not frequent attenders the subsequent year. This highlights a typical complex-systems feature of unpredictability at the individual level but predictability at the systems level.

Practically, this suggests that frequent attendance at EDs is not a difficulty with few, consistent individual high users but rather a stable feature of the system as a whole. Consequently, interventions aimed at individual frequent attenders would be unlikely to have any long-term positive impact on the problem of frequent attendance per se. This is because any frequent attenders successfully targeted by individual-level interventions would simply be replaced by new frequent attenders. To address issues around frequent attendance, system capacity, access, and efficiency, system-level interventions should therefore be considered.

A number of other projects have linked the CUREd data to external data sources. These have included 1) linking in Mental Health Trust data to understand patterns of attendance at urgent care by patients with serious mental illness, 2) linking with hospice data to describe the use of the ED by patients known to be palliative and hence understand how to preempt these events, and 3) linking with specific patient groups, such as those with functional neurologic disorders, to describe the impact of interventions aimed at reducing symptoms.

## Discussion

The immense amount of routinely collected data currently held within health and social care settings internationally provides huge opportunities for researchers to address key health and social care challenges. These relate to how patients access care, how care is delivered, and what the outcomes are for patients and services. This provides invaluable insights into what can be improved, whether this is particular services, interventions, or patient groups where signals in the data infer inequity in access and outcomes that can be addressed. The use of routine data provides economies of scale, which is potentially attractive to research funders, and also provides the opportunity to increase the generalizability of findings.

This article has described the development of a unique research platform in the United Kingdom, linking together NHS data from 1 region with a population of 5.6 million. Data were linked from telephone advice service, ambulance service, ED, and acute hospital admissions data. The article outlines the regulatory approvals and the process of acquiring and linking the data for research. It then describes some of the analyses undertaken and the opportunities for different methodological approaches to establishing causation in research. The analyses described in this article provide some indication of the power that such large routine data sets can have in demonstrating how patients navigate services and what their outcomes are. In the examples given, it can be seen how identification of patient groups might be important when planning interventions to be more appropriate for patients and improve efficiency and clinical outcomes. For example, in describing patterns of frequent users within the system, we have identified that interventions are better targeted at the system level rather than the individual patient. This is because frequent users are not always the same patients over a number of years but are being replaced by new patients all the time. When trying to provide better access for parents of young children, we have established that there are particular gaps for those younger than 5 y, where other services are not available when needed. Finally, we have shown how transfers in care from one service to another (in this case telephone helpline services) result in inaccuracies in the triage decisions, which lead to significant numbers being referred to the wrong level of care.

There is an increasing recognition by research funding bodies globally that accessing routine data for research is vital. One of the greatest challenges is establishing the data set itself. Often, this is not considered “research,” and yet the development, regulation, curation, and production of research-ready data is considerably time-consuming and expensive. Identifying the investment for such developments can be challenging. In addition, the expertise required to undertake the development of research-ready data sets is a field difficult to recruit to within health-related research. Further investment is needed in infrastructure for data set development and expertise.

### Limitations

The greatest barrier to linking individual patient activity was insufficient identifiable information captured by the included ambulance service. This was especially problematic for ambulance service activity, which did not involve attendance on scene (e.g., dealt with via telephone, “hear and treat”). Failure to identify records belonging to the same patient will have inflated the number of “distinct individuals” in CUREd. The opposite problem is also possible, with individuals sharing a date of birth, postcode, and gender potentially (in the absence of other information) being considered the same person (this likely affects twins in childhood greatest). Both of these issues are also present in other data sources (e.g., NHS Hospital Episode Statistics). Although NHS number and date of birth were used as the gold standard for linkage, the provision and use of NHS numbers is not perfect, with some individuals receiving more than 1 NHS number and care sometimes being accessed under another individual’s NHS number.^19^

Some patients will have been conveyed by the included ambulance service to hospitals or other facilitates from which we did not receive data, either because they were outside of the Yorkshire and Humber region or because they were not an acute hospital (e.g., mental health units). Other patients will have been treated and conveyed by other ambulance services from which we did not collect data; this is especially true of the Humber area, which is primarily served by a different ambulance service. However, ambulance services routinely attend incidents outside their primary areas (especially near area borders) for operational reasons.

We set no exclusion criteria for emergency or urgent care activity; even if it was not possible to link patients’ pathways, their care activity was still recorded. However, a reconciliation of monthly ED attendances reported to CUREd compared with NHS Digital (under the A&E Commissioning Dataset return) revealed Trusts did not supply wholly consistent data to both, with some failing to report activity to CUREd and at other times failing to report activity to NHS Digital. This often presented as a total or near total absence of activity for a period of time.

Data supplied by some data providers were corrupted in the data extraction process through the use of unsuitable software tools. The most common corruptions were loss of precision due to the inappropriate conversion of large integers into scientific notation and the loss of meaningful leading zeros. Although the former could be identified using validation rules, the latter (due to poor practices in the creation of some national coding standards) was not always possible to identify. However, we believe this data corruption is more likely to occur in ad hoc extraction processes, and such problems are also present in data reported to NHS Digital.^20^

### Future Considerations

Our experiences to date have highlighted the potential power that routine data can provide for researchers. In describing the development of the data platform from acquiring the approvals, through to obtaining data and then processing it, we hope to have outlined the challenges and skill required. It is important that as researchers, methodologists, and data specialists, we ensure that knowledge in how to deliver research-ready routine data is shared. Making the use of routine data commonplace will require partnership between the collectors, owners, and guardians of the data (health and social care providers, national data providers, e.g., NHS Digital and others), industry partners to apply effective digital technology to deliver solutions, and health data users, including data analysts, health services researchers, and health and social care providers.

Data acquisition from providers can be challenging, and this often involves a negotiation that takes several weeks, as further requests need processing. However, these obtaining these data frequently results in partial data sets that require linkage with the previously supplied data. The development of trusted research environments for research purposes offers a solution to the time delays and lengthy communications required on an individual provider basis to acquire accurate data. Federating data within trusted research environments can lead to a more secure and efficient use of data by a larger group of researchers. Inevitably, this means that the opportunities to exploit such data for improving health care delivery will be greater. It also provides the opportunity to work with data providers to ensure that the data delivered is more consistent and of higher quality. Developing and sharing meta-data specifications would also enhance the development of real-world data research. By sharing meta-data catalogs that can allow researchers and data providers to share common standards, the quality of data sets will improve. Examples of such meta-data catalogs are found in the Health Data Science UK Innovation Gateway (https://www.healthdatagateway.org/), which aims to provide a repository of data sets and related tools for researchers, data custodians, patients, and the public.

### Summary

This article has provided an overview of the development of a linked data platform for research. It has described how the data set evolved and was established and some of the key outputs delivered. It has also discussed future opportunities researchers and funders should embrace, current limitations, and future requirements to ensure this important area of research flourishes.
